# Subcutaneous immunoglobulins for the treatment of a patient with antisynthetase syndrome and secondary chronic immunodeficiency after anti-CD20 treatment: a case report

**DOI:** 10.1186/s13256-017-1211-9

**Published:** 2017-03-04

**Authors:** Patrick Cherin, Christophe de Jaeger, Jean-Charles Crave, Jean-Christophe Delain, Abir Tadmouri, Zahir Amoura

**Affiliations:** 1 Department of Internal Medicine, Pitié-Salpetrière Hospital Group, 47-83 Boulevard de l’Hôpital, 75013 Paris, France; 2 Institut Galliera, 4 rue de Galliera, 75016 Paris, France; 3 Octapharma France SAS, Boulogne Billancourt, France; 4 ClinSearch, Malakoff, France

**Keywords:** Antisynthetase syndrome, Myositis, Subcutaneous human immunoglobulin, Secondary immunodeficiency, Anti-Jo-1 antibody, Autoimmune disease, Case report

## Abstract

**Background:**

Antisynthetase syndrome is a rare and debilitating multiorgan disease characterized by inflammatory myopathy, interstitial lung disease, cutaneous involvement, and frequent chronic inflammation of the joints. Standard treatments include corticosteroids and immunosuppressants. In some cases, treatment resistance may develop. Administration of immunoglobulins intravenously is recommended in patients with drug-resistant antisynthetase syndrome.

**Case presentation:**

Here, we describe the case of a 56-year-old woman of Algerian origin. She is the first case of a patient with multidrug-resistant antisynthetase syndrome featuring pulmonary involvement and arthropathy, and chronic secondary immune deficiency with recurrent infections, after anti-CD20 treatment, in which her primary antisynthetase syndrome-related symptoms and secondary immune deficiency were treated successfully with subcutaneous administration of immunoglobulin. The administration of immunoglobulin subcutaneously was introduced at a dose of 2 g/kg per month and was well tolerated. Clinical improvement was observed within 3 months of initiation of subcutaneous administration of immunoglobulin. After 22 months of treatment, she showed a significant improvement in terms of muscle strength, pulmonary involvement, arthralgia, and immunodeficiency. Her serum creatine phosphokinase and C-reactive protein levels remained normal. Finally, she was compliant and entirely satisfied with the treatment.

**Conclusions:**

Taken together, these observations suggest that administration of immunoglobulin subcutaneously may be a useful therapeutic approach to tackle steroid-refractory antisynthetase syndrome while ensuring minimal side effects and improved treatment compliance. This treatment also allowed, in our case, for the regression of the chronic immunodeficiency secondary to rituximab treatment.

## Background

Antisynthetase syndrome (aSS) is a rare idiopathic autoimmune condition occurring in a subgroup of patients with polymyositis and dermatomyositis who are positive for one or several of eight anti-aminoacyl transfer ribonucleic acid (RNA) synthetase (ARS) auto-antibodies [1]. Six major clinical hallmarks define the syndrome: fever, myositis, interstitial lung disease, “mechanic’s hands”, Raynaud phenomenon, and inflammatory polyarthritis [2]. Symptoms may occur individually or in a variety of combinations; hence, a straightforward diagnosis is challenging [3, 4]. Of interest, there is evidence that the clinical picture and outcome of aSS are intimately tied to the identity of the ARS antibody being expressed [5, 6]. The most common form of aSS is anti-Jo-1 antibody-associated (anti-histidyl-transfer RNA synthetase) and features polymyositis of proximal muscles alongside interstitial lung disease or, rarely, pulmonary hypertension [7–9].

Due to multiorgan involvement, aSS is a debilitating condition associated with increased morbidity and mortality, especially when pulmonary function is affected [10, 11]. Moreover, myocardial complications and malignancies may also occasionally be observed within this patient population and contribute to poor prognosis [12–14].

Currently, glucocorticoids are the mainstay of therapy and may be completed by immunosuppressive treatments, typically methotrexate (MTX) or azathioprine, in order to decrease steroid dose and to achieve disease control [15–17]. Cyclophosphamide can be used to control interstitial lung disease. In treatment-refractory patients, rituximab (RTX) may also be considered [18, 19]. Because of the low prevalence of aSS, there is a lack of randomized controlled trials comparing the efficacy and safety of different treatment approaches. However, a few studies supported administration of immunoglobulins intravenously (IVIg) as a promising therapeutic avenue for treatment-refractory patients, or those wishing to avoid the risks associated with chronic corticosteroid exposure [16, 20–23]. More recently, high-dose administration of immunoglobulin subcutaneously (SCIg) has arisen as a less invasive and more economical alternative to IVIg [20, 24, 25].

Here, we report the case of a patient with aSS, refractory to steroid and immunosuppressive treatment, and poorly tolerating both RTX and IVIg. She developed secondary chronic immune deficiency with recurrent infections after anti-CD20 (RTX) treatment. In this patient, a combined SCIg and MTX treatment significantly improved her aSS-specific symptoms and overall health status which, in addition, enabled the disappearance of secondary immune deficiency.

## Case presentation

A 56-year-old woman, 70 kg, of Algerian origin was referred to us in August 2003 presenting with fatigue, proximal and bilateral muscular weakness (muscle testing score of 69 points compared to a score of 88 in healthy individuals), apprehension to grasp, and difficulties in getting dressed. Additional symptoms included effort dyspnea, swollen hands, and purple erythema of her eyelids. Appendicitis, sciatica, tachycardia, hypertension, and asthma were listed in her medical history. Her creatine phosphokinase (CPK, muscle enzymes) levels were six times the normal (N) level. Auto-antibodies measurements were initially not performed.

A muscle biopsy was performed, and showed characteristic patterns of dermatomyositis with perifascicular atrophy, evidence of injury to capillaries and perifascicular myofibers, and inflammatory infiltrates in the perimysial region (predominantly CD4+). She was diagnosed as having dermatomyositis in November 2003 and prednisone treatment (1 mg/kg per day) was initiated. A repeated search for malignancy was negative. A diagnosis of a mild interstitial pneumonitis together with the presence of anti-Jo1 antibodies further confirmed the suspicion of aSS. Her gamma globulin levels were normal.

Since treatment response was incomplete, immunosuppressant therapy with azathioprine (2 mg/kg per day), which was replaced after 9 months by MTX (15 mg per week), was introduced. However, both were poorly tolerated and she developed cytopenia. Therefore, infusions with IVIg (2 g/kg per month) were initiated for six months, in addition to steroids.

In September 2006, due to lack of response to these different therapies, RTX (2 g every 6 months) was introduced and our patient reported an improvement in her articular and muscular pain. Yet, because of the development of hypogammaglobulinemia, RTX was discontinued in October 2011. Of importance, no immune deficit had been present prior to the introduction of RTX.

**Fig. 1:**
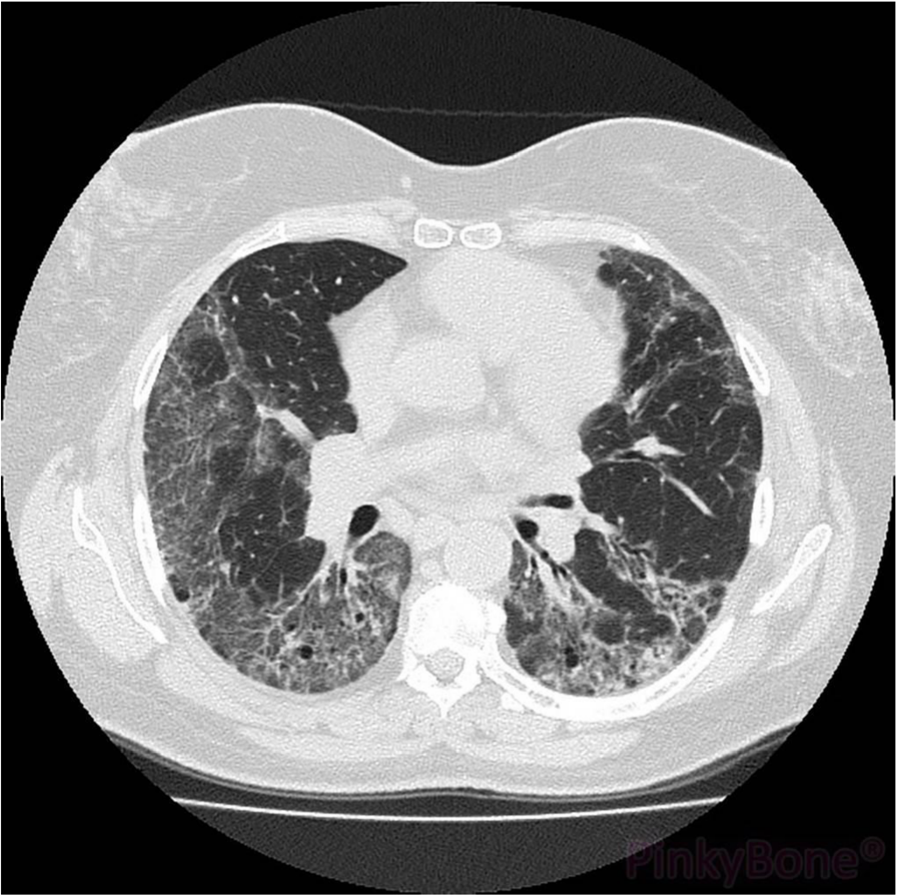
Lung scan showing the presence of interstitial basal lung infiltrate

She was readmitted in December 2012 with a muscle weakness score of 75.5 points (over 88 points in healthy individuals) [21]. Her CPK levels were normal, but probing for anti-Jo-1 antibody was positive. In addition, a lung scan revealed the presence of interstitial basal lung infiltrate (Fig. 1). Testing of pulmonary function showed a reduction of single-breath diffusion capacity for carbon monoxide (DLCO; 48%), with a total lung capacity (TLC) of 72%, and forced expiratory volume in 1 second (FEV_1_)/vital capacity (VC) at 73%. A slight muscle inflammation of her thighs was also evidenced by magnetic resonance imaging (MRI). In terms of treatment strategy, a bolus injection of IVIg (2 g/kg) was administered, but she developed adverse effects, including headache and distal paresthesia, after which she refused to receive another IVIg infusion. Her treatment adherence was poor and in addition to refusing to try a different IVIg formulation, she had also resumed the corticosteroid treatment in November 2012. In fact, she expressed wishes against hospitalizations and stopped all the treatments.

An immunologic evaluation showed hypogammaglobulinemia (4.4 g/l), with reduced total immunoglobulin G (IgG) (4.2 g/l), and a reduction in each subclass of IgG: IgG1 2.48 g/l (N >3.82 g/l), IgG2 1.65 g/l (N >2.41), IgG3 0.14 g/l (N >0.2), and IgG4 0.038 g/l (N >0.18 g/l). Her IgA (0.5 g/l, N >0.7) and IgM levels (0.34 g/l, N >0.4) were also decreased. Her IgE levels were normal.

**Fig. 2:**
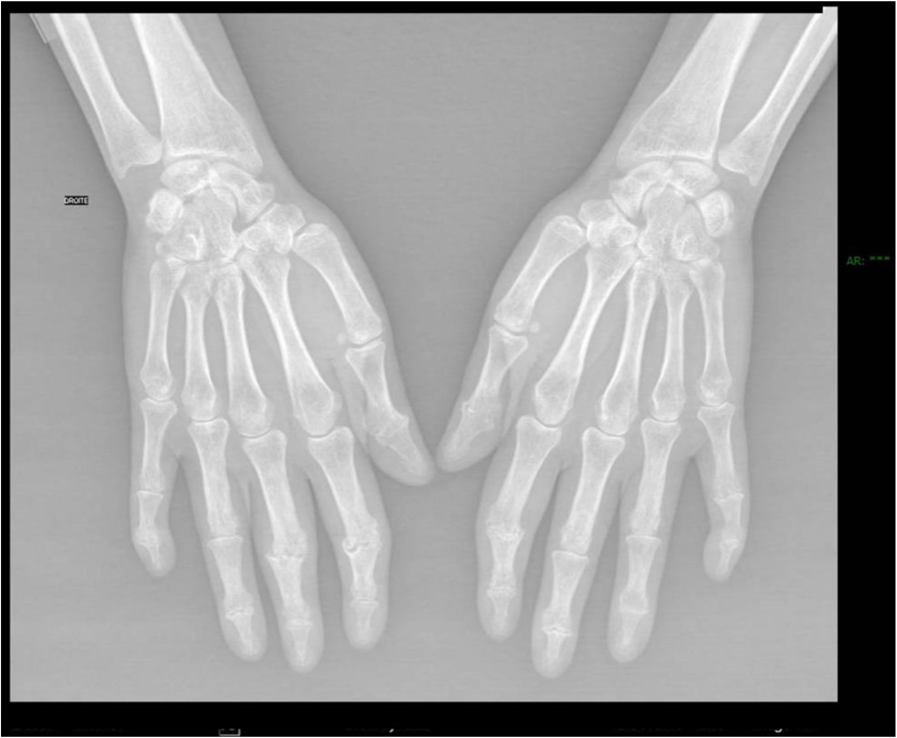
Magnetic resonance imaging scanner of the right hand showing an advanced and destructive arthropathy associated with significant synovitis of the proximal interphalangeal joints of the first, second, and third rays and, to a lesser extent, of the fourth and fifth rays

She was seen again in May 2014, when her general health had deteriorated due to the discontinuation of steroids, RTX, and IgG treatment. In particular, she complained about severe fatigue and presented with swelling of the proximal interphalangeal joints of her right index and middle finger. An MRI scan of her right hand revealed an advanced and destructive arthropathy associated with significant synovitis of the proximal interphalangeal joints of the first, second, and third rays and, to a lesser extent, of the fourth and fifth rays (Fig. 2). Her metacarpal phalangeal joints were not affected, but an erosive synovitis of the dorsal scapholunate articulation and beginning erosion of the lunate were observed. Her radioulnar joint was not affected.

At this point, her muscle weakness score was 70/88, with a muscle strength of +3 as measured bilaterally at her middle trapezius, major gluteal, and psoas muscles. Her muscle disability was rated 18/75 (score ranging from 0 no disability to 75 maximum disability) [22]. An MRI of her thighs did not reveal any significant evolution of myopathy since the previous examination in 2012. In particular, there was no important inflammatory signal of the muscle and no sign of amyotrophy.

Likewise, a thorax scan confirmed the stabilization of the abnormalities reported in November 2012, with mostly the basal regions being affected. Pulmonary function testing was also indicative of overall stability compared to 2012: single-breath DLCO was 46%, TLC 70%, and FEV_1_/VC 73%. Whereas DLCO, TLC (−30%), and functional respiratory deficit (25%) were unchanged, a slight decrease of forced expiratory flow (FEF)_25–75_ was noted.

Of importance, she complained of frequent infections of the upper airways, including pharyngitis, sinusitis, bronchitis, and otitis for the past 2 years. Consistent with these recurrent infections, an immunologic evaluation confirmed a persistent hypogammaglobulinemia (5.0 g/l), with reduced total IgG (4.1 g/l), and reduction of each subclass of IgG: IgG1 2.3 g/l, IgG2 1.48 g/l, IgG3 0.11 g/l, and IgG4 0.04 g/l. This picture was evocative of a persistent common variable immunodeficiency (CVID) secondary to RTX. Her C-reactive protein (CRP) level was 22.7 mg/l and her CPK level was 489 UI/l.

Treatment with Gammanorm® (SCIg) was initiated in July 2014 (2 g/kg per month divided into two infusions per week). After 3 and 6 months, respectively, of treatment, she reported diffuse pain, which was present since discontinuation of Cortancyl® (prednisone), and general fatigue. Arthritis of her proximal and metacarpal phalangeal joints was still present, but her muscle weakness score had improved to 75/88 and muscle disability was rated 12/75 [22]. Of importance, no novel infectious episode was reported. In fact, Gammanorm® (SCIg) was well tolerated and she confirmed a slight improvement in her general health state.

In January 2015, MTX (15 mg/week) was reintroduced and a significant clinical improvement was achieved by April. Notably, her serum CRP (3.3 mg/l) and CPK (63 UI/l) returned to normalized levels despite persisting fatigue, along with arthritis of her metacarpal and proximal interphalangeal joints (arthritis confirmed at a consultation in May 2015). Nevertheless, the normalization of her immunodeficiency and the significant improvement in the state of her general health remained stable, as reassessed in November 2015.

Finally, in May 2016, joint manifestations had disappeared and fatigue had regressed significantly. No infection had been observed during the past 18 months. Her muscle weakness score had also improved to 82/88 and muscle disability was rated 9/75. Her CRP and CPK levels were normal. As shown by immunologic evaluation, hypogammaglobulinemia had resolved and all subclasses of IgG were normalized.

Moreover, the results of a thorax scan indicated a slight improvement in basal lung infiltrate, alongside pulmonary function testing: DLCO of 55%, TLC of 75%, and a FEV_1_/VC 74%.

## Discussion

To the best of our knowledge, this is the first report of a case of aSS pathology with concomitant secondary immune deficiency secondary to RTX, successfully treated with SCIg. Of importance, and in addition to a progressive improvement of aSS-related symptoms, our patient fully recovered from RTX-induced immune deficiency, tolerated SCIg treatment very well, and reported a substantial increase in her quality of life. Hence, SCIg emerges as a safe, efficient, and patient-friendly alternative for the treatment of this rare, complex, and burdensome form of polymyositis.

Although rare, aSS is a challenging pathology because of the complexity and severity of symptoms [3, 4], some of which are highly debilitating and/or life threatening [10–13]. Unfortunately, the most common therapeutic avenue – corticoid treatment – is jeopardized by side effects and the development of treatment resistance. In the present case, a first-line corticoid therapy did not achieve satisfactory disease control. Conversely, a number of second-line treatments, including immunosuppressant therapy (azathioprine, MTX, RTX) and IVIg, although somewhat efficient in terms of symptomatology were poorly tolerated by the patient. Notably, anti-CD20 immunosuppressant therapy provoked a persistent immunodeficiency, including hypogammaglobulinemia. Due to the recurrence of heavy side effects combined with poor therapeutic efficacy, our patient’s compliance with the treatment degraded and she became reluctant to try alternative approaches (for instance, different IVIg formulations) [26, 27].

Under the double burden of an untreated aSS and a persistent immune deficiency, our patient’s general health dramatically deteriorated. In this precarious situation, the introduction of SCIg (2 g/kg per month) proved to be efficacious in eradicating the immune deficiency and progressively downscaling aSS-related symptoms. In fact, 6 months into the treatment, her immune function had recovered, as proven by the absence of new infectious episodes and the normalization of her IgG and CRP levels. Her pulmonary and muscle function had also improved and her CPK normalized. In addition, the general improvement in her health enabled resumption of her MTX treatment, and eventually led to full disease control, including alleviation of arthropathy, regression of fatigue, and a substantial increase in quality of life within less than 12 months of SCIg initiation.

We believe that this double action of SCIg on stabilization of immune function and aSS disease management is of particular interest in the light of several recent reports featuring the prolonged effects of RTX on the immune system. In fact, in their retrospective chart review, Kaplan *et al*. identified a subset of patients receiving RTX treatment and presenting with prolonged immune suppression, which required immunoglobulin replacement therapy even after discontinuation of anti-CD20 treatment [28].

Likewise, Levy *et al*. report in their retrospective analysis of 32 studies conducted in France between 2001 and 2014 three cases of prolonged hypogammaglobulinemia in patients who had received RTX as a second-line treatment for immune thrombocytopenia [29]. Of interest, the clinical picture was reminiscent of CVID, including recurrent infections, and lasted up to 2 years after RTX discontinuation. A further analysis of the literature allowed the authors to conclude that severe hypogammaglobulinemia is a rare but severe and long-lasting side effect of RTX treatment. These reports, together with our case, call for caution and the close monitoring of immune function in patients who receive RTX for the treatment of autoimmune disorders.

In addition to proving efficient at tackling both aSS pathology and secondary immunodeficiency, SCIg was also particularly well tolerated in the present case. Given the poor tolerance of a variety of previous therapies and our patient’s growing opposition to treatment, this represents a chief clinical benefit. Our observations are consistent with previous reports, indicating that the subcutaneous route of Ig administration is generally better tolerated than the systemic one [30]. Another crucial advantage of the SCIg formulation over IVIg is of course the ease of administration, which allows for home-based use and thereby reduces costly hospitalizations, while increasing patient autonomy [31].

## Conclusions

In summary, the case reported here supports SCIg as an efficient and safe alternative, which is more economical than IVIg, for the treatment of complex and/or refractory forms of aSS. Of importance, in this particular case, the clinical benefit of SCIg went beyond the control of primary aSS-related symptoms, since a secondary immune deficiency was also successfully treated. In the light of these promising outcomes, we believe that SCIg should also be considered a valuable tool for the management of other autoimmune conditions.
